# Comparing the Self- and External Assessment Versions of the HCL-33 as Screening Instruments for Bipolar Disorder in Older Depressed Patients

**DOI:** 10.3389/fpsyt.2021.727992

**Published:** 2021-11-15

**Authors:** Xinqiao Zhang, Wen Li, Na Zhao, Yu Jin, Teris Cheung, Gabor S. Ungvari, Xi-Ling Cui, Gang Wang, Yu-Tao Xiang, Jules Angst

**Affiliations:** ^1^The National Clinical Research Center for Mental Disorders and Beijing Key Laboratory of Mental Disorders, Beijing Anding Hospital and the Advanced Innovation Center for Human Brain Protection, School of Mental Health, Capital Medical University, Beijing, China; ^2^Shanghai Key Laboratory of Forensic Medicine, Key Laboratory of Forensic Science, Shanghai Forensic Service Platform, Academy of Forensic Science, Ministry of Justice, Shanghai, China; ^3^Unit of Psychiatry, Department of Public Health and Medicinal Administration, Institute of Translational Medicine, Faculty of Health Sciences, University of Macau, Taipa, Macao SAR, China; ^4^Centre for Cognitive and Brain Sciences, University of Macau, Macao, Macao SAR, China; ^5^Institute of Advanced Studies in Humanities and Social Sciences, University of Macau, Taipa, Macao SAR, China; ^6^College of Education for the Future, Beijing Normal University, Beijing, China; ^7^School of Nursing, Hong Kong Polytechnic University, Hung Hom, Hong Kong SAR, China; ^8^Division of Psychiatry, School of Medicine, University of Western Australia/Graylands Hospital, Perth, WA, Australia; ^9^University of Notre Dame Australia, Fremantle, WA, Australia; ^10^Department of Business Administration, Hong Kong Shue Yan University, North Point, Hong Kong SAR, China; ^11^Zurich University Psychiatric Hospital, Zurich, Switzerland

**Keywords:** bipolar disorder, HCL-33, older adults, major depressive disorder, psychometric property

## Abstract

**Objectives:** The misdiagnosis of bipolar disorder (BD) as major depressive disorder (MDD) is common in depressed older adults. The self-rated HCL-33 and its external assessment version (HCL-33-EA) have been developed to screen for hypomanic symptoms. This study compared the screening ability of these two instruments to discriminate BD from MDD.

**Methods:** A total of 215 patients (107 with BD and 108 with MDD) and their carers were recruited. Patients and their carers completed the HCL-33 and HCL-33-EA, respectively. The consistency of the total score and the positive response to each item between the two scales was calculated with the intraclass correlation coefficient (ICC) and Cohen's kappa coefficient separately. Receiver operating characteristics (ROC) curves were drawn for both instruments. The optimal cut-off points were determined according to the maximum Youden's Index. The areas under the ROC curve (AUC) of the HCL-33 and HCL-33-EA were calculated separately and compared. The sensitivity and specificity at the optimal cut-off values were also calculated separately for the HCL-33 and HCL-33-EA.

**Results:** The intraclass correlation coefficient (ICC) between the total scores of the HCL-33 and HCL-33-EA was 0.823 (95% CI = 0.774–0.862). The positive response rate on all items showed high agreement between the two instruments. ROC curve analysis demonstrated that the total scores of both HCL-33 and HCL-33-EA differentiated well between MDD and BD, while there was no significant difference in the AUCs between the two scales (Z = 0.422, *P* = 0.673). The optimal cutoff values for the HCL-33 and HCL-33-EA were 14 and 12, respectively. With the optimal cutoff value, the sensitivities of the HCL-33 and HCL-33-EA were 88.8% and 93.5%, and their specificities were 82.4% and 79.6%.

**Conclusion:** Both the HCL-33 and HCL-33-EA had good screening ability for discriminating BD from MDD in depressed older adults.

## Introduction

With the improvement of healthcare services in the past decades, many patients with bipolar disorder (BD) live on into older adulthood. The diagnosis of BD is associated with increased health service use and premature mortality in older adults (1). The prevalence of BD in this population varied greatly between different studies, ranging from 0.1% in the community to 8–10% in psychiatric hospitals (2).

Patients with BD are frequently misdiagnosed in clinical practice, in a range of 48% (3) to 69% of cases (4). The misdiagnosis of BD is also common in older adults, although the rate seems to decrease with age (5). Older BD patients, particularly those with BD-type II (BD-II) and BD-not otherwise specified (BD-NOS), were most often misdiagnosed as having major depressive disorder (MDD) (6). The misdiagnosis of BD as MDD could be partly attributable to the unawareness and underreporting of hypomanic symptoms, since patients with BD tend to seek medical help during their depressive but rarely during their hypomanic episodes, when they often enjoy the elevated mood (4). In addition, the course of BD often starts with a depressive episode and may even be followed by predominantly depressive episodes for a considerable period of time (7–9). The time gap between the first depressive episode and the subsequent first manic/hypomanic episode is longer in older than in younger patients: for example, this gap was 17 years in BD patients aged 60 years and above, while the corresponding figure was only 3.5 years in those aged 40 and below (10). The late appearance of a manic/hypomanic episode in older BD patients increases the likelihood of their BD being misdiagnosed as MDD. Late recognition of BD results in delayed, inadequate, and inappropriate treatment (11, 12).

Regular screening for hypomania facilitates the timely diagnosis of BD. In the past decades, both clinician-administered and self-rated screening instruments have been developed to screen for hypomania. Structured clinical interviews, such as the Structured Clinical Interview for the Diagnostic and Statistical Manual of Mental Disorders, 5th Edition (SCID-5) (13) and the Composite International Diagnostic Interview (CIDI) (14), represent the most reliable and valid approach for diagnosing BD, but they are time-consuming and need to be administered by skilled clinicians (15, 16). Several self-report measures have therefore been developed to screen for BD, including the 33-item Hypomania Checklist (HCL-33) (17). The HCL-33, a modified version of the 32-item Hypomania Checklist (HCL-32) (18), a widely-used self-report instrument to screen for hypomania, has been validated in depressed Chinese adults (17). Recently, a parallel external-assessment version of the HCL-33 (HCL-33-EA) has been constructed for patients' carers, family members and friends (19). Carers are familiar with patients' mood swings and daily lives. Moreover, cognitive, hearing, and visual problems, that are common in older patients, may hinder the use of self-report scales.

The clinical features of BD in older patients are different from those in their younger counterparts (2), making it important to validate the HCL-33 and the HCL-33-EA in an older sample. Our study examined the screening ability of the HCL-33 and HCL-33-EA to differentiate BD from MDD and evaluated the consistency of the screening ability of the two instruments.

## Methods

### Participants

The study was conducted in the geriatric psychiatry department of Beijing Anding Hospital, a major tertiary psychiatric hospital in China, between July 2017 and November 2019. Patients attending the geriatric psychiatry department were consecutively invited to participate in the study if they were (1) aged 60 years old and above; (2) experiencing a depressive episode; (3) diagnosed with MDD or BD according to the 10th Revision of the International Statistical Classification of Diseases and Related Health Problems (ICD-10); (4) accompanied by at least one caregiver. The depressive episode and diagnoses of BD and MDD were initially established by the patients' treating psychiatrists and confirmed by a research psychiatrist using the Chinese version of the Mini International Neuropsychiatric Interview (MINI), Version 5.0, based on the Diagnostic and Statistical Manual of Mental Disorders, 4th Edition (DSM-IV) (20, 21). The exclusion criteria were comorbid psychiatric disorders and severe medical or neurological conditions. Carers of each patient were also invited to participate in the survey without any exclusion criteria. The study protocol was approved by the Clinical Research Ethics Committee of Beijing Anding Hospital. All participants provided written informed consent.

### Instruments

The demographic characteristics of patients and carers and patients' clinical features were collected. The HCL-33, Chinese version, is a self-administered and validated questionnaire (17). The HCL-33-EA is based on the original version of the HCL-33 and was administered to patients' carers (family members and close friends) to assess hypomanic symptoms. Both scales consist of 33 items with dichotomous responses of “yes/no,” comprehensively covering various aspects of hypomania. The total score on each scale is obtained by adding up all positive responses and ranges from 0 to 33, with a higher total score representing more severe hypomanic symptoms. In this study, all patients were asked to complete the HCL-33 and their carers the HCL-33-EA.

### Statistical Analysis

All the analyses were performed using the Statistical Package for Social Sciences (SPSS), Version 20.0 and the Mecalc software. The normality of continuous variables was examined with the P–P plot. The paired sample *t* tests and Wilcoxon Signed Rank tests were used to compare the total scores of the HCL-33 and HCL-33-EA, as appropriate. The frequency of positive responses for items of the two scales was compared between BD and MDD patients using chi-square tests. The intraclass correlation coefficient (ICC) was employed to assess the consistency between the total scores of the two scales, while the Cohen's kappa coefficient was used to assess the consistency between the positive response to each item of the two scales with a Cohen's kappa coefficient value of “0–0.20” considered as slight, “0.21–0.40” as fair, “0.41–0.60” as moderate, “0.61–0.80” as substantial, and “0.81–1.00” as almost perfect agreement (22).

A previous study (23) found a two-factor structure for the HCL-33, comprising “active/elated” (items 2–6, 8, 10–15, 17–19 and 21–27) and “substance use/indulging” (items 28, 29 and 30) factors. Principal components analysis (PCA) was used to examine the factor structure of the HCL-33-EA. As recommended previously (23), items were allocated to a specific factor when their loading value was > 0.4.

The sensitivity and specificity at each possible cutoff value of the HCL-33 and HCL-33-EA for discriminating BD from MDD were calculated using the receiver operating-characteristics (ROC) curve analysis with the MINI diagnosis as the gold standard. The discriminating ability was examined with the area under the ROC curve (AUC) where the AUC of >0.6 indicated acceptable discrimination (24). The optimal cut-off value was determined according to the Youden's Index, which was the maximum of summation of sensitivity and specificity at each cut-off value (25). The pairwise comparison of the ROC curves of the HCL-33 and HCL-33-EA was conducted using the DeLong method (26). The consistency between the HCL-33 and the HCL-33-EA was tested using Cohen's kappa with <0.40 signaling poor agreement, 0.40–0.75 fair to good agreement, and >0.75 excellent agreement (27). Significance level was set at *P* < 0.05 (two-side) in all analyses.

## Results

### Demographic Characteristics of the Total Sample

In total, 232 patients were screened and invited to participate in our study; 17 (7.3%) refused or failed to complete the interview. Eventually, 215 patients were included, with 108 diagnosed with MDD and 107 with BD [71 with type I BD (BD-I) and 36 with BD-II]. The mean age of the patients was 67.3 [standard deviation (SD) = 5.5] years. All patient carers completed the assessment (*n* = 215); their mean age was 49.1 (SD = 14.4) years. Most patient carers were offspring (59.1%, *n* = 127), followed by spouses (34.9%, *n* = 75), siblings (2.8%, *n* = 6), and other relations (2.8%, *n* = 7). The mean length of cohabitation with the patients was 30.3 (SD = 10.2) years.

### Total Scores of the HCL-33 and the HCL-33-EA

The mean total score on the HCL-33 was 12.6 (SD = 7.2) in the whole sample, and 7.5 (SD = 5.9), 17.7 (SD = 4.0), 18.2 (SD = 3.9), and 16.8 (SD = 4.0) in patients with MDD, BD, BD-I, and BD-II, respectively. The mean total score on the HCL-33-EA was 12.5 (SD = 7.3) in the whole sample, and 7.4 (SD = 5.7), 17.7 (SD = 4.6), 18.4 (SD = 4.1), and 16.3 (SD = 5.1) in patients with MDD, BD, BD-I, and BD-II, respectively. There were no significant differences between the total scores on HCL-33 and HCL-33-EA in the whole sample (*P* = 0.759), or in patients with MDD (*P* = 0.920), BD (*P* = 0.557), BD-I (*P* = 0.587), or BD-II (*P* = 0.724) (Table 1).

**Table 1:** Comparison between the total score of the HCL-33 and HCL-33-EA.

**Sub-group**	**Sample size (*n*)**	**HCL-33**		**HCL-33-EA**		**Statistics^a^**	
		**Mean**	**SD**	**Mean**	**SD**	***Z***	***P***
MDD	108	7.5	5.9	7.4	5.7	−0.101	0.920
BD	107	17.7	4.0	17.7	4.6	−0.587	0.557
BD-I	71	18.2	3.9	18.4	4.1	−0.543	0.587
BD-II	36	16.8	4.0	16.3	5.1	−0.353	0.724
Total sample	215	12.6	7.2	12.5	7.3	−0.307	0.759

a *Wilcoxon Signed Rank tests*.

*BD, bipolar disorder; BD-I, type I bipolar disorder; BD-II, type II bipolar disorder; HCL-33, the 33-item Hypomania Checklist; HCL-33-EA, the 33-item Hypomania Checklist-external assessment; MDD, major depressive disorder; SD, standard deviation*.

### Positive Responses to the Individual Items of the HCL-33 and the HCL-33-EA

**Figure 1:**
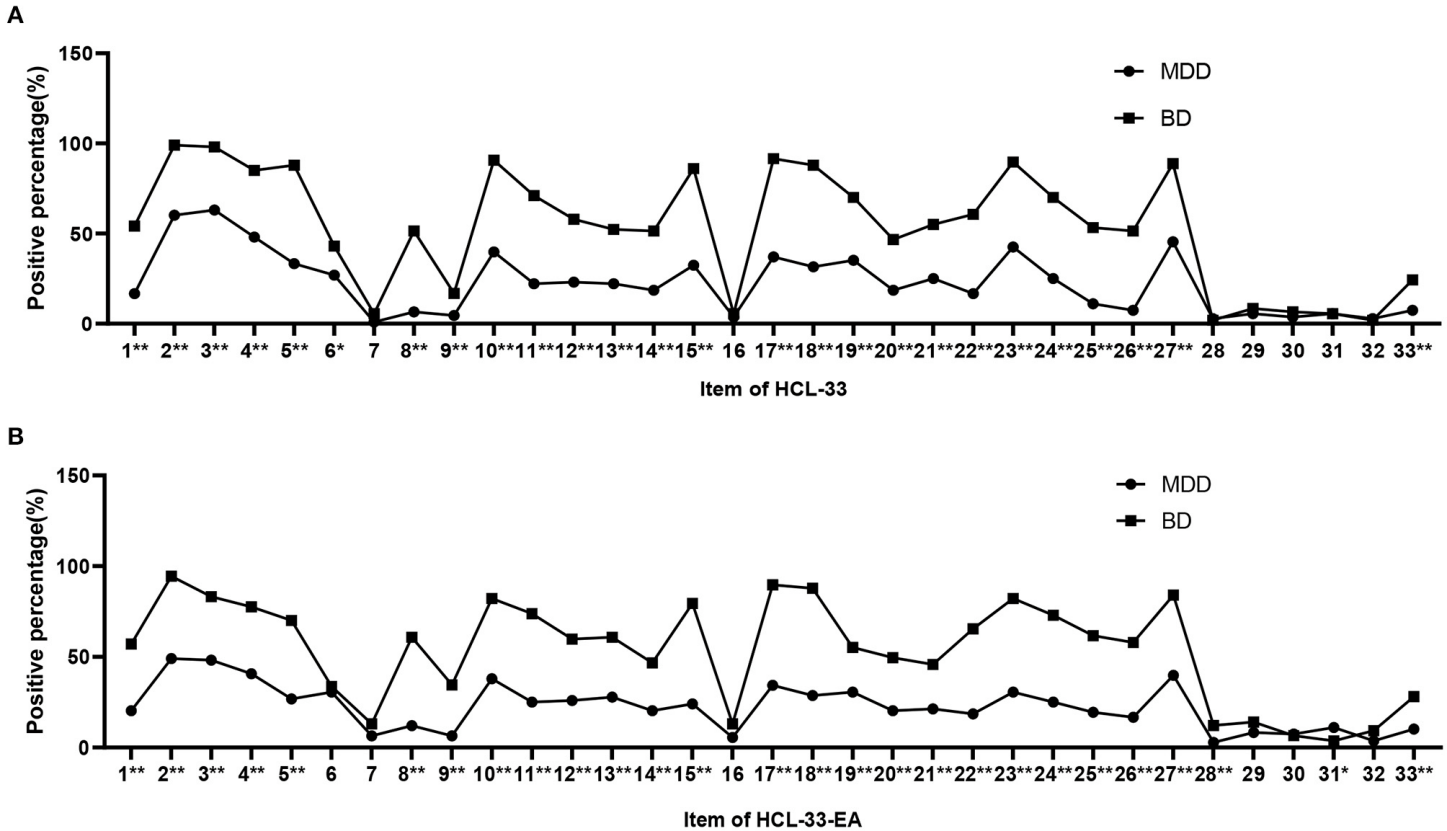
Positive responses for items of the HCL-33 and HCL-33-EA. **(A)** Positive responses for items of the HCL-33 in patients with MDD and BD. **(B)** The positive responses for items of the HCL-33-EA in patients with MDD and BD. *P < 0.05; **P < 0.01; BD, bipolar disorder; MDD, major depressive disorder.

Positive responses to the HCL-33 items in patients with BD were significantly more frequent than in patients with MDD except for items 7, 16, and 28–32 (Figure 1A). The same was true for the HCL-33-EA except for items 6, 7, 16, 29, 30, and 32 (Figure 1B).

### Consistency Between the HCL-33 and the HCL-33-EA

The ICC between the total scores of the HCL-33 and the HCL-33-EA was 0.823 [95% confidence interval (CI) = 0.774–0.862] in the whole sample and was 0.846 (95% CI = 0.766–0.900), 0.815 (95% CI = 0.747–0.866), and 0.672 (95% CI = 0.217–0.887) in patients cared for by their spouses, offspring, and others, respectively.

The percentages of the consistent screening results of the HCL-33 and HCL-33-EA items measured by the kappa coefficient were higher than 70%. The consistency of the positive response was substantial between the items 11, 17, and 18 of the two scales (κ = 0.61–0.80, *P* < 0.01), was fair (κ = 0.21–0.40, *P* < 0.01) between the items 7, 16, 31, 32, and 33, was slight (κ = 0.16, *P* < 0.01) between items 28, and was moderate (κ = 0.41–0.60, *P* < 0.01) between the remaining items (Table 2).

**Table 2:** Consistency between the rating results of each item of the HCL-33 and HCL-33-EA.

**Item**		**Percentage of agreement (%)**	**Cohen's kappa**
Item 1	He/She needs less sleep	79.07	0.55^**^
Item 2	He/She is more energetic and more active	82.79	0.54^**^
Item 3	He/She is more self-confident	78.60	0.47^**^
Item 4	He/She enjoys his/her work more	77.67	0.53^**^
Item 5	He/She is more sociable (makes more phone calls, goes out more)	76.74	0.54^**^
Item 6	He/She wants to travel and/or does travel more	75.81	0.46^**^
Item 7	He/She tends to drive faster or take more risks when driving	91.63	0.32^**^
Item 8	He/She spends more money/too much money	85.12	0.66^**^
Item 9	He/She takes more risks in daily life (in his/her work and/or other activities)	84.65	0.43^**^
Item 10	He/She is physically more active (sport, etc.)	79.07	0.55^**^
Item 11	He/She plans more activities or projects	85.12	0.70^**^
Item 12	He/She has more ideas, is more creative	74.42	0.47^**^
Item 13	He/She is less shy or inhibited	69.77	0.38^**^
Item 14	He/She wears more colorful and more extravagant clothes/make-up	77.21	0.49^**^
Item 15	He/She wants to meet or actually does meet more people	78.60	0.57^**^
Item 16	He/She is more interested in sex and/or is more sexually active	89.77	0.22^**^
Item 17	He/She talks more	83.72	0.65^**^
Item 18	He/She thinks faster	81.86	0.63^**^
Item 19	He/She makes more jokes or puns when talking	76.28	0.53^**^
Item 20	He/She is more easily distracted	74.42	0.43^**^
Item 21	He/She engages in lots of new things	74.88	0.46^**^
Item 22	His/Her thoughts jump from topic to topic	77.21	0.53^**^
Item 23	He/She is more impatient and/or gets irritable more easily	80.00	0.58^**^
Item 24	He/She is more impatient and/or gets irritable more easily	77.21	0.54^**^
Item 25	He/She can be exhausting or irritating for others	80.47	0.58^**^
Item 26	He/She gets into more quarrels	80.93	0.57^**^
Item 27	His/Her mood is higher, more optimistic	80.00	0.57^**^
Item 28	He/She drinks more coffee	92.09	0.16^**^
Item 29	He/She smokes more cigarettes	93.02	0.58^**^
Item 30	He/She drinks more alcohol	93.49	0.43^**^
Item 31	He/She takes more drugs (sedatives, anxiolytics, stimulants…)	90.70	0.24^**^
Item 32	He/She games or gambles more	93.95	0.29^**^
Item 33	He/She eats more or binges more	80.47	0.33^**^

** *P < 0.01*.

### Factor Analysis of the HCL-33-EA

To explore the factor structure of HCL-33-EA, the Kaiser-Meyer-Olkin (KMO) Measure of Sampling Adequacy and the Bartlett's test of sphericity were performed (28, 29), giving a KMO of 0.869 and χ^2^ of 2,452.828 (*P* < 0.01), which indicates that the study sample was adequate and suitable for PCA. Nine factors with an eigenvalue greater than 1 emerged and cumulatively explained 60.4% of the total variance (Supplementary Figure 1). The first three factors had eigenvalues of 8.7, 2.1, and 1.9, respectively, and explained 38.2% of the total variance. Factor I consisted of 20 items (items 2–5, 8–15, 17–19, 21–24, and 27) and could be characterized as “active/elated”, Factor II consisted of 2 items (items 25 and 26) and could be characterized as “irritable”, and Factor III consisted of 6 items (items 7, 28-30, 32, and 33) and could be characterized as “substance use/indulging” (Table 3). Few items loaded on other factors with eigenvalues > 1, making them difficult to characterize. A three-factor structure was ultimately established.

**Table 3:** Factor loadings of the HCL-33-EA.

**Items**	**HCL-33-EA**		
	**Factor I**	**Factor II**	**Factor III**
Item 1	0.388	0.235	0.042
Item 2	**0.713**	−0.212	−0.072
Item 3	**0.610**	−0.322	−0.200
Item 4	**0.579**	−0.287	−0.137
Item 5	**0.587**	−0.311	−0.042
Item 6	0.261	−0.321	0.153
Item 7	0.190	0.095	**0.415**
Item 8	**0.601**	0.253	0.076
Item 9	**0.478**	0.218	0.193
Item 10	**0.671**	−0.127	−0.076
Item 11	**0.712**	−0.144	−0.021
Item 12	**0.612**	−0.130	0.108
Item 13	**0.495**	−0.042	0.222
Item 14	**0.425**	−0.162	0.213
Item 15	**0.676**	−0.194	−0.027
Item 16	0.245	0.278	0.164
Item 17	**0.698**	0.001	−0.191
Item 18	**0.758**	−0.103	−0.176
Item 19	**0.450**	−0.269	0.009
Item 20	0.383	0.351	−0.048
Item 21	**0.543**	−0.088	0.132
Item 22	**0.565**	0.263	−0.079
Item 23	**0.682**	−0.059	−0.106
Item 24	**0.529**	0.473	−0.206
Item 25	0.465	**0.614**	−0.230
Item 26	0.441	**0.475**	−0.339
Item 27	**0.701**	−0.010	−0.025
Item 28	0.233	0.039	**0.427**
Item 29	0.270	0.240	**0.479**
Item 30	0.080	0.270	**0.512**
Item 31	−0.052	0.049	0.172
Item 32	0.156	−0.182	**0.492**
Item 33	0.379	0.097	**0.402**

*Bolded values: factor loading >0.4*.

### ROC Curves Analyses for the HCL-33 and HCL-33-EA

**Figure 2:**
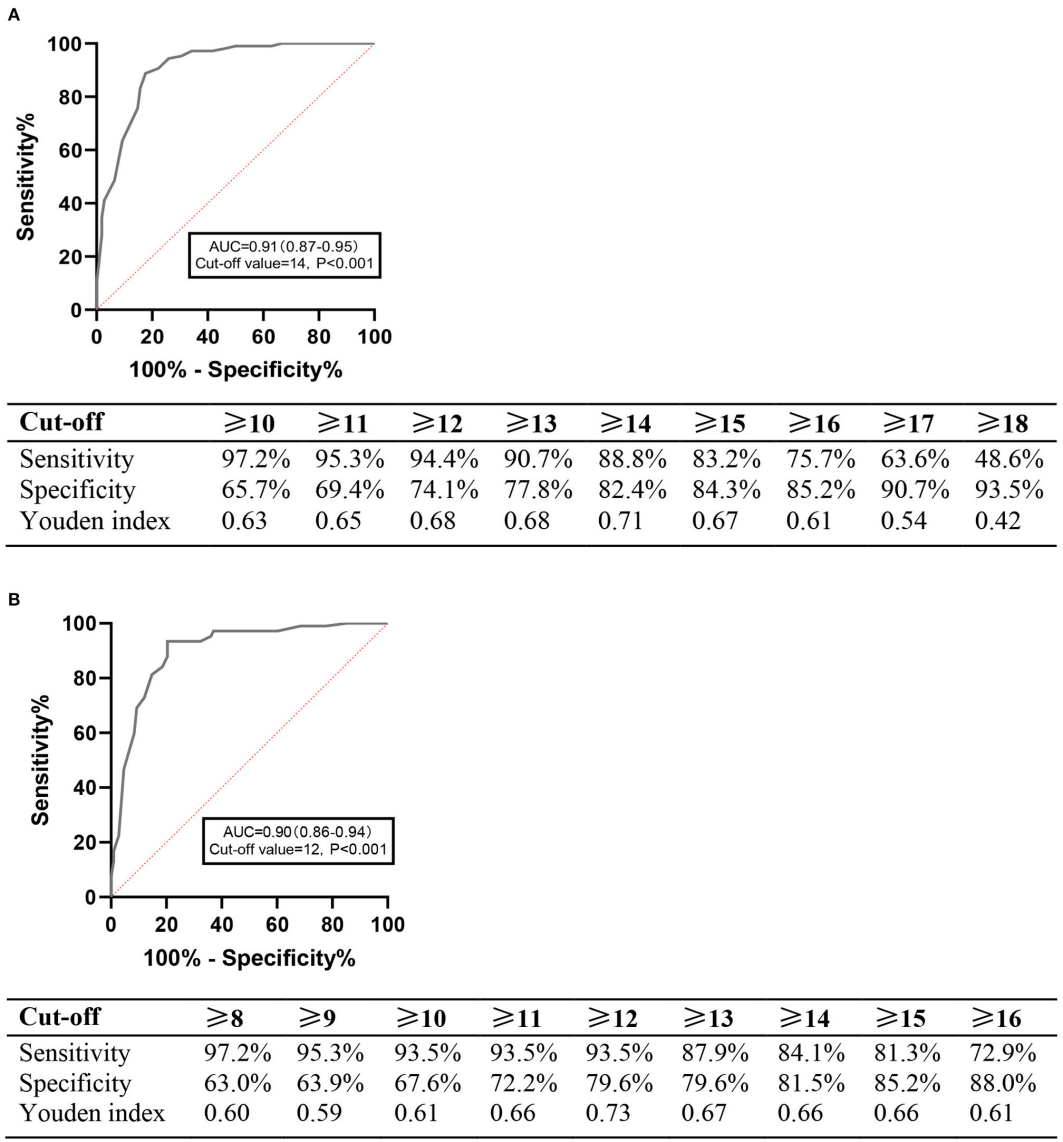
ROC analyses. **(A)** ROC curve for the HCL-33. **(B)** ROC curves for the HCL-33-EA.

ROC curve analysis demonstrated that the HCL-33 total score could differentiate well between MDD and BD, with the AUC of 0.91 (95% CI=0.87–0.95). The optimal cut-off point was 14, with a Youden index of 0.71, and the corresponding sensitivity and specificity figures were 88.8 and 82.4%, respectively (Figure 2A).

ROC curve analysis also demonstrated that the HCL-33-EA total score could differentiate well between MDD and BD, with the AUC of 0.90 (95% CI = 0.86–0.94). The optimal cutoff value was 12, with a Youden index of 0.73, and the corresponding sensitivity and specificity figures were 93.5 and 79.6%, respectively (Figure 2B). There was no significant difference between the AUC of the HCL-33 and the HCL-33-EA (Z = 0.422, *P* = 0.673).

### Kappa Coefficients of the HCL-33 and HCL-33-EA

Using the optimal cutoffs of 14 for the HCL-33 and 12 for the HCL-33-EA in the sample, the consistency of the HCL-33 and the HCL-33-EA was fairly good (kappa coefficient = 0.737, *P* < 0.001).

## Discussion

To the best of our knowledge, this is the first study to compare the screening consistency between the self-rated and external assessment versions of the HCL-33 in depressed older adults. The ICC between the HCL-33 and HCL-33-EA total scores was 0.823, which is similar to the finding in depressed younger adults (Spearman's *r* = 0.46) (19). The consistency of the total scores on the two instruments was higher in patients cared for by their spouses (ICC = 0.846, 95% CI = 0.766–0.900), followed by those cared for by offspring (ICC = 0.815, 95% CI = 0.747–0.866), and others (ICC = 0.672, 95% CI = 0.217–0.887), probably because spouses were more familiar with the patients' mood swings than other carers.

The positive responses to all the 33 items showed sufficient agreement between the two HCL scales, with most of the items achieving moderate agreement (κ > 0.4). This is slightly different from the findings of a study conducted in adult patients (30), which found insufficient agreement between the HCL-33 and HCL-33-EA in 6 of the 33 items. The present findings indicate high consistency between the items of the HCL-33 and HCL-33-EA in older depressed Chinese patients. In addition, the three-factor structure of the HCL-33-EA differed from the two-factor structure as reported previously for the HCL-33 (23). More specifically, although the same 19 items (items 2–5, 8, 10–15, 17–19, 21–24, and 27) loaded on Factor I of both scales, items 25 and 26 loaded on Factor I of the HCL-33 but on Factor II of the HCL-33-EA. Moreover, three items (28–30) that loaded on Factor II of the HCL-33 loaded on Factor III of the HCL-33-EA together with three further items (7, 32 and 33). Inconsistencies were also found between previous studies on the HCL-32 and HCL-33, including two-factor (17, 18, 31–33), three-factor (34–37) and four-factor structures (38, 39). The discrepancy between studies could be partly due to different study characteristics (e.g., age, gender and severity of illness) and types of rater (e.g., patients for the HCL-33 vs. patients' carers for the HCL-33-EA).

The ROC curve analysis revealed that both the HCL-33 and HCL-33-EA total scores differentiated well between MDD and BD in older adults. The optimal cutoff value for the HCL-33 was 14 in this study, which is similar to the cutoff value of 15 found in Chinese adult patients (17). The sensitivity and specificity at the optimal cutoff value in our study were higher than those reported in adult patients (sensitivity: 88.8 vs. 62%; specificity 82.4 vs. 74%) (17). The optimal cutoff value for the HCL-33-EA total score was 12, with a higher sensitivity (93.5%) and a lower specificity (79.6%) than for the HCL-33, suggesting that carers could be more sensitive in recognizing hypomanic symptoms than the patients themselves. Since this was the first study examining the screening efficacy of the HCL-33-EA, direct comparisons with previous studies could not be made.

This study did not find any significant difference between the AUC of the two instruments (Z = 0.422, *P* = 0.673), which suggests that the HCL-33 and HCL-33-EA have similar ability to discriminate BD from MDD in depressed older patients. In addition, the kappa coefficients between the two instruments showed that the consistency was fairly good. A similar finding was reported in a Chinese adult sample, in which the total scores of HCL-33 and HCL-33-EA were significantly and positively correlated (19). The two instruments could therefore be interchangeable in clinical practice to discriminate BD from MDD in older Chinese patients.

The study has several limitations that need to be acknowledged. First, the sample size was relatively small, which may have decreased the statistical power of the findings. Second, due to the single study site, the sample could not represent depressed older adults from other regions in China. Third, patients with comorbid psychiatric disorders, severe medical or neurological conditions were excluded from the study, which further limits the generalizability of the findings.

In conclusion, both the HCL-33 and the HCL-33-EA showed satisfactory psychometric properties in discriminating BD from MDD in depressed older adults, while the consistency of the discriminative ability of the two scales was also comparable.

## Data Availability Statement

The datasets presented in this article are not readily available because the Clinical Research Ethics Committee of Beijing Anding Hospital that approved the study prohibits the authors from making publicly available the research dataset of clinical studies. Readers and all interested researchers may contact Y-TX (Email address: xyutly@gmail.com) for details. Y-TX could apply to the Clinical Research Ethics Committee of Beijing Anding Hospital for the release of the data. Requests to access the datasets should be directed to xyutly@gmail.com.

## Ethics Statement

All participants provided written informed consent. The study protocol was reviewed and approved by the Clinical Research Ethics Committee of Beijing Anding Hospital.

## Author Contributions

Y-TX and GW: study design. XZ, WL, NZ, and YJ: data collection, analysis, and interpretation. XZ, WL, TC, GW, and Y-TX: drafting of the manuscript. GU, X-LC, and JA: critical revision of the manuscript. All authors contributed to the article and approved the submitted version.

## Funding

This study was supported by the National Science and Technology Major Project for investigating new drugs (2018ZX09201-014), the Beijing Municipal Science and Technology Commission (No. Z181100001518005), and the University of Macau (MYRG2019-00066-FHS).

## Conflict of Interest

The authors declare that the research was conducted in the absence of any commercial or financial relationships that could be construed as a potential conflict of interest.

## Publisher's Note

All claims expressed in this article are solely those of the authors and do not necessarily represent those of their affiliated organizations, or those of the publisher, the editors and the reviewers. Any product that may be evaluated in this article, or claim that may be made by its manufacturer, is not guaranteed or endorsed by the publisher.

## References

[1] Sajatovic M Strejilevich SA Gildengers AG Dols A Shulman KI. A report on older-age bipolar disorder from the International Society for Bipolar Disorders Task Force. Bipolar Disord. (2015) 17:689–704. 10.1111/bdi.12331 26384588 PMC4623878

[2] Vasudev A Thomas A. ‘Bipolar disorder’ in the elderly: What's in a name? Maturitas. (2010) 66:231–5. 10.1016/j.maturitas.2010.02.013 20307944

[3] Lish JD Dime-Meenan S Whybrow PC Price RA Hirschfeld RMA. The National Depressive and Manic-depressive Association (DMDA) survey of bipolar members. J Affect Disord. (1994) 31:281–94. 10.1016/0165-0327(94)90104-X 7989643

[4] Hirschfeld RM Lewis L Vornik LA. Perceptions and impact of bipolar disorder: how far have we really come? Results of the national depressive and manic-depressive association 2000 survey of individuals with bipolar disorder. J Clin Psychiatry. (2003) 64:161–74. 10.4088/JCP.v64n0209 12633125

[5] Kessing LV. Diagnostic stability in bipolar disorder in clinical practise as according to ICD-10. J Affect Disord. (2005) 85:293–9. 10.1016/j.jad.2004.11.001 15780699

[6] Takeshima M Kurata K. Late-life bipolar depression due to the soft form of bipolar disorder compared to unipolar depression: an inpatient chart review study. J Affect Disord. (2010) 123:64–70. 10.1016/j.jad.2009.07.019 19716179

[7] Cha B Kim JH Ha TH Chang JS Ha K. Polarity of the first episode and time to diagnosis of bipolar I disorder. Psychiatry Investig. (2009) 6:96–101. 10.4306/pi.2009.6.2.96 20046381 PMC2796048

[8] Judd LL Akiskal HS Schettler PJ Endicott J Maser J Solomon DA. The long-term natural history of the weekly symptomatic status of bipolar I disorder. Arch Gen Psychiatry. (2002) 59:530–7. 10.1001/archpsyc.59.6.530 12044195

[9] Solomon DA Leon AC Maser JD Truman CJ Coryell W Endicott J. Distinguishing bipolar major depression from unipolar major depression with the screening assessment of depression-polarity (SAD-P). J Clin Psychiatry. (2006) 67:434–42. 10.4088/JCP.v67n0315 16649831

[10] Broadhead J Jacoby R. Mania in old age: a first prospective study. Int J Geriatr Psychiatry. (1990) 5:215–22. 10.1002/gps.930050403

[11] Nasrallah HA. Consequences of misdiagnosis: inaccurate treatment and poor patient outcomes in bipolar disorder. J Clin Psychiatry. (2015) 76:e1328. 10.4088/JCP.14016tx2c 26528666

[12] Post RM Leverich GS Kupka RW Keck PE Jr. McElroy SL Altshuler LL. Early-onset bipolar disorder and treatment delay are risk factors for poor outcome in adulthood. J Clin Psychiatry. (2010) 71:864–72. 10.4088/JCP.08m04994yel 20667291

[13] First MB Williams JB Karg RS Spitzer RL. User's Guide for the SCID-5-CV Structured Clinical Interview for DSM-5® Disorders: Clinical Version. Arlington, VA: American Psychiatric Publishing, Inc (2016).

[14] Kessler RC Üstün TB. The world mental health (WMH) survey initiative version of the world health organization (WHO) composite international diagnostic interview (CIDI). Int J Methods Psychiatr Res. (2004) 13:93–121. 10.1002/mpr.168 15297906 PMC6878592

[15] Akiskal HS. Classification, diagnosis and boundaries of bipolar disorders: a review. Bipolar disorder. (2002) 5:1–96. 10.1002/047084650X.ch118199232

[16] Miller CJ Johnson SL Eisner L. Assessment tools for adult bipolar disorder. Clin Psychol. (2009) 16:188–201. 10.1111/j.1468-2850.2009.01158.x 20360999 PMC2847794

[17] Feng Y Xiang YT Huang W Wang G Feng L Tian TF. The 33-item Hypomania Checklist (HCL-33): a new self-completed screening instrument for bipolar disorder. J Affect Disord. (2016) 190:214–20. 10.1016/j.jad.2015.09.057 26519642

[18] Angst J Adolfsson R Benazzi F Gamma A Hantouche E Meyer TD. The HCL-32: towards a self-assessment tool for hypomanic symptoms in outpatients. J Affect Disord. (2005) 88:217–33. 10.1016/j.jad.2005.05.011 16125784

[19] Fang M Wang Y-Y Feng Y Ungvari GS Ng CH Wang G. Exploration of the psychometric properties of the 33-item Hypomania Checklist—external assessment (HCL-33-EA). J Affect Disord. (2019) 245:987–90. 10.1016/j.jad.2018.11.023 30699884

[20] Si TM Shu L Dang W Su Y-A Chen JX Dong WT. Evaluation of the reliability and validity of Chinese version of the Mini-International Neuropsychiatric Interview in patients with mental disorders. Chin Mental Health J. (2009) 23:493–503.

[21] Sheehan D Lecrubier Y Harnett-Sheehan K Janavs J Weiller EHT Hergueta T. The Mini-International Neuropsychiatric Interview (M.I.N.I.): the development and validation of a structured diagnostic psychiatric interview for DSM-IV and ICD-10. J Clin Psychiatry. (1998) 59(Suppl 20):22–33;quiz 4. 9881538

[22] Landis JR Koch GG. The measurement of observer agreement for categorical data. Biometrics. (1977) 33:159–74. 10.2307/2529310 843571

[23] Zhang X Li W Zhao N Cheung T Ungvari GS Wang G. Use of the 33-Item Hypomania Checklist (HCL-33) to distinguish bipolar disorder from major depressive disorder in older adults. J Geriatric Psychiatry Neurol. (2021) 2021:8919887211016065. 10.1177/08919887211016065 34044653

[24] Yang S Berdine G. The receiver operating characteristic (ROC) curve. Southwest Respir Critic Care Chron. (2017) 5:34–6. 10.12746/swrccc.v5i19.391

[25] Youden WJ. Index for rating diagnostic tests. Cancer. (1950) 3(1):32–5. 10.1002/1097-0142(1950)3:1<32::AID-CNCR2820030106>3.0.CO;2-3 15405679

[26] DeLong ER DeLong DM Clarke-Pearson DL. Comparing the areas under two or more correlated receiver operating characteristic curves: a nonparametric approach. Biometrics. (1988) 44:837–45. 10.2307/2531595 3203132

[27] Fleiss JL. Statistical Methods for Rates and Proportions. New York, NY: Wiley. (1981).

[28] Cerny BA Kaiser HF. A study of a measure of sampling adequacy for factor-analytic correlation matrices. Multivariate Behav Res. (1977) 12:43–7. 10.1207/s15327906mbr1201_3 26804143

[29] Kaiser HF. An index of factorial simplicity. Psychometrika. (1974) 39:31–6. 10.1007/BF02291575

[30] Lojko D Dudek D Angst J Siwek M Michalak M Rybakowski J. The 33-item Hypomania Checklist (HCL-33)—a study of the consistency between self—and external assessments in Polish bipolar patients. Psychiatr Pol. (2016) 50:1085–92. 10.12740/PP/66358 28211548

[31] Huang X Liu W Feng B Tan Q Ji J. Applicability of the Chinese version of the Hypomania Symptom Checklist (HCL-32) scale for outpatients of psychiatric departments in general hospitals. PLoS ONE. (2013) 8:e75631. 10.1371/journal.pone.0075631 24116062 PMC3792138

[32] Bech P Christensen EM Vinberg M Bech-Andersen G Kessing LV. From items to syndromes in the Hypomania Checklist (HCL-32): psychometric validation and clinical validity analysis. J Affect Disord. (2011) 132:48–54. 10.1016/j.jad.2011.01.017 21349588

[33] Wu Y-S Angst J Ou C-S Chen H-C Lu R-B. Validation of the Chinese version of the Hypomania Checklist (HCL-32) as an instrument for detecting hypo(mania) in patients with mood disorders. J Affect Disord. (2008) 106:133–43. 10.1016/j.jad.2007.06.004 17644185

[34] Yang HC Yuan CM Liu TB Li LJ Peng HJ Liao CP. Validity of the 32-item Hypomania Checklist (HCL-32) in a clinical sample with mood disorders in China. BMC Psychiatry. (2011) 11:84. 10.1186/1471-244X-11-84 21575151 PMC3112081

[35] Holtmann M Portner F Duketis E Flechtner HH Angst J Lehmkuhl G. Validation of the Hypomania Checklist (HCL-32) in a nonclinical sample of German adolescents. J Adolesc. (2009) 32:1075–88. 10.1016/j.adolescence.2009.03.004 19328541

[36] Yoon BH Angst J Bahk WM Wang HR Bae SO Kim MD. Psychometric properties of the hypomania checklist-32 in Korean Patients with mood disorders. Clin Psychopharmacol Neurosci. (2017) 15:352–60. 10.9758/cpn.2017.15.4.352 29073747 PMC5678485

[37] Prieto ML Youngstrom EA Ozerdem A Altinbas K Quiroz D Aydemir O. Different patterns of manic/hypomanic symptoms in depression: a pilot modification of the hypomania checklist-32 to assess mixed depression. J Affect Disord. (2015) 172:355–60. 10.1016/j.jad.2014.09.047 25451437

[38] Glaus J Van Meter A Cui L Marangoni C Merikangas KR. Factorial structure and familial aggregation of the Hypomania Checklist-32 (HCL-32): results of the NIMH family study of affective spectrum disorders. Compr Psychiatry. (2018) 84:7–14. 10.1016/j.comppsych.2018.03.010 29655654 PMC6002901

[39] Haghighi M Bajoghli H Angst J Holsboer-Trachsler E Brand S. The Farsi version of the hypomania check-list 32 (HCL-32): applicability and indication of a four-factorial solution. BMC Psychiatry. (2011) 11:14. 10.1186/1471-244X-11-14 21251272 PMC3036591

