# Supplementary figures and images for: Comparing the Self- and External Assessment Versions of the HCL-33 as Screening Instruments for Bipolar Disorder in Older Depressed Patients
Source: Front Psychiatry. 2021 Nov 15;12:727992. doi: 10.3389/fpsyt.2021.727992 (PMC8634141; doi:10.3389/fpsyt.2021.727992)

**Supplementary Figure 1. Scree Plot for the HCL-33-EA**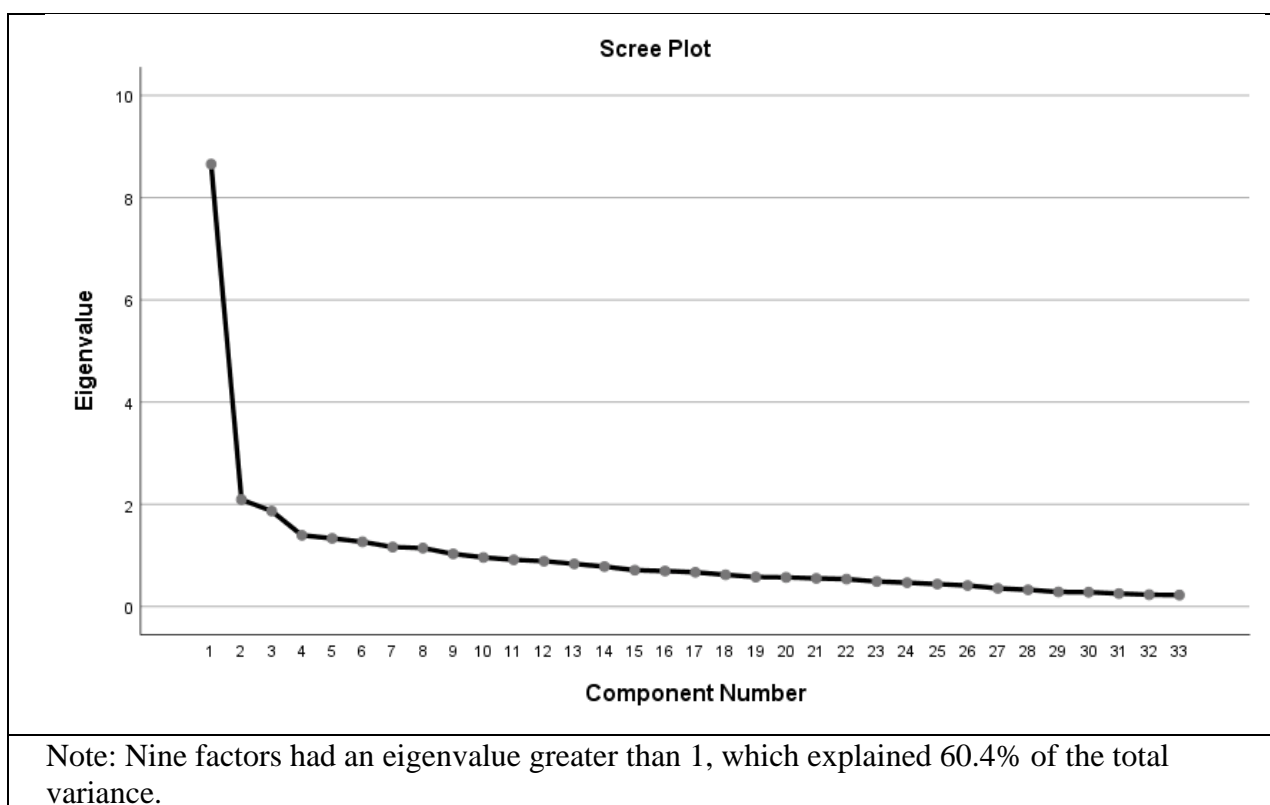

Supplement: Supplementary file 1 [file Data_Sheet_1.pdf]
